# Dynamics of Water and Other Molecular Liquids Confined Within Voids and on Surface of Lignin Aggregates in Aging Bio Crude Oils

**DOI:** 10.3389/fchem.2021.753958

**Published:** 2021-12-17

**Authors:** Massimo Bonini, Emiliano Fratini, Antonio Faraone

**Affiliations:** ^1^ Department of Chemistry “Ugo Schiff” and CSGI, University of Florence, Sesto Fiorentino, Italy; ^2^ Center for Neutron Research, National Institute of Standards and Technology, Gaithersburg, MD, United States

**Keywords:** pyrolysis oil, lignin aggregation, neutron scattering, water dynamics, confined water, lignin dynamics

## Abstract

Neutron scattering methods were employed to study the microscopic structure and dynamics of Bio Crude Oils (BCOs) and their lignin fractions. The structure of the carbonaceous aggregates was investigated using Small Angle Neutron Scattering to reveal a fractal hierarchy as well as a growth of the aggregates as the aging of the BCO proceeds. Elastic Neutron Scattering measurements indicate that BCO liquid phase, comprised of water and other hydrogenated molecular liquids, is in a state of extreme confinement. Quasi-Elastic Neutron Scattering yields information on the molecular motions, indicating that long range translational diffusion is suppressed and only localized dynamics take place on the tens of picosecond time range. The obtained results provide quantitative information on the molecular activity, as aging proceed, in these reactive materials of relevance as potential renewable energy sources.

## 1 Introduction

The energetic scenario depicted by most analysts and Kyoto agreement on CO_2_ emissions control require the development of new fuels based on renewable sources. In the last couple of decades, Bio Crude Oils (BCOs) have attracted a considerable attention as possible renewable energy from biomass (Ringer et al., 2006). These oils are obtained from the pyrolysis of biomass: i.e., by heating a feed-stock such as wood, agricultural wastes, paper, algae, animal wastes, etc., at high temperature, and rapidly quenching the obtained liquid products. Unfortunately, BCOs are poorly stable since numerous reactions take place after their production, making them very reactive and leading to several problems in their handling and final use. In particular, compared to conventional mineral fuels, pyrolysis oils show long-term instability with a strong dependency on the storage temperature (Oasmaa and Kuoppala, 2003). Therefore, the characterization of the BCOs and the way they are affected by aging represents a crucial step in order to employ these oils in practical applications and in particular as substitutes of mineral oils. In general, BCOs are formed by a large number of organic compounds, mainly carboxylic acids, carbohydrates and lignin derivatives, together with a variable amount of water. Unfortunately, some of these organic compounds are very reactive and seem to be the main responsible for the aging process (Bridgwater, 2008). During storage these components chemically react to produce larger molecules leading to changes in the physical properties, such as viscosity and density. Previous studies proposed that etherification and esterification occurring between hydroxyl, carbonyl, and carboxyl groups (Czernik et al., 1994) are the main chemical reactions taking place in pyrolysis oils and producing water as a byproduct of the condensation reactions. Lignin’s derived compounds are indicated as pyrolytic lignin and are obtained as the water-insoluble fraction of BCOs (SipiläSipila et al., 1998). Gel Permeation Chromatography and ^13^C-NMR measurements (Scholze et al., 2001) demonstrated that pyrolytic lignin mainly consist of trimers and tetramers, even though larger structural units remain intact during the pyrolysis. These results are in agreement with the thermal ejection theory formulated by Piskorz (Overend and Chornet, 1999), where lignin oligomers are considered to be directly expelled from wood particles as a result of a partial cracking of lignin molecules during the pyrolysis. In a previous SANS investigation, some of the Authors have verified the validity of this theory and elucidated the role of pyrolytic lignin in the aging of BCOs (Fratini et al., 2006). In particular, the possibility to correlate the aggregation between pyrolytic lignin into clusters with the evolution of the BCOs chemical and physical properties was disclosed. This is of great importance in the formulation of pyrolysis fuels with long-term stability. The investigation reported an increase of both the volume fraction of the scattering objects and the number of spherical sub-units per cluster (i.e., the aggregation number) as time passes after BCO production. The obtained fractal dimensions values range between 1.4 and 1.5, i.e., values typical of branched structures that are generated by the aggregation of small lignin units as a consequence of the chemical reactivity of this peculiar fluid (Fratini et al., 2006).

The microscopic dynamics of the liquid phases in the BCO plays a crucial role in determining the reactions taking place during aging. Dynamic neutron scattering techniques offer the unique capability to measure the single particle dynamics of the hydrogen atoms in the system over length scales of the order of the Ångstrom and time scales ranging from few picoseconds to nanoseconds. This microscopic mobility of the liquid species is indicative of the activity in the system.

Among the liquid species present in BCO, water accounts for more than 20% (see Table 1). However, the environment the water molecules experience is vastly different from the bulk: in fact, water is most reasonably confined on the surface and within the nanoscopic voids of the carbonaceous aggregates generated by the lignin units as well as intermixed and hydrogen bonded with other molecular liquids (carboxylic acids, aldehydes/ketones, ethers, esters, etc.) (Oasmaa et al., 2003). As such, water in BCO should be considered interfacial water and an exemplary case of how, in many instances of relevance, water molecules are found confined on surfaces, in microcavity, or dispersed within a different liquid phase.

**TABLE 1:** Physico-chemical parameters of the BCO from BTG (Chiaramonti et al., 2011). Here mass% indicates the percentage mass fraction.

Parameter	Value
Carbon (mass%)	42.79
Hydrogen (mass%)	7.57
Nitrogen (mass%)	<0.01
Oxygen (balance) (mass%)	49.57
Water content (mass%)	21.7
Ash content (mass%)	0.047
Solids content (mass%)	0.27
Density (kg/m^3^)	1207
Kinematic viscosity (10^–6^ m^2^/*s*)	65.1
pH	2.85

The present work reports the results of an investigation, performed using mainly Quasi-Elastic Neutron Scattering (QENS) (Bée, 1988; Gardner et al., 2020), of the microscopic mobility of the hydrogen atoms in BCO as a function of aging. In parallel, Small Angle Neutron Scattering (SANS) is employed to determine the micro-structure of the aggregates in BCO and its evolution during aging. The analysis of the temperature dependence of the mean squared displacement of the hydrogen atoms provides information on the confinement experienced by the liquid phases. The analysis of QENS data provides information on the nanoscale dynamics in terms of both time scale and length scale of the mobility. A comparison with the dynamics of the diverse lignin fractions is carried out as well.

## 2 Methods

### 2.1 Samples

Bio-crude oil was supplied by Biomass Technology Group (BTG, Netherlands) and was produced in their fast pyrolysis plant in Enschede from a batch of pine chips. Main physico-chemical properties of the investigated BCO are reported in Table 1. Additional details on this batch can be found elsewhere (Chiaramonti et al., 2011). Samples corresponding to four aging stages have been investigated and are labelled, hereinafter, as “fresh,” “12 months,” “12 months at 40°C” and “15 months.” All of the samples were stored at room temperature during the first month after their production. After the first month, “fresh” sample was frozen and taken back to the liquid state before carrying out the measurements. Similarly, “12 months” sample was stored at room temperature and frozen 13 months after production (12 months after “fresh”). Sample labeled as “15 months” was never frozen and always stored at room temperature, as the measurements were carried out exactly 16 months after production. Sample labeled as “12 months at 40°C” was stored, after the first month at room temperature, at 40°C during the next year and then frozen until measurements. Samples containing low and high molecular weight lignin fractions have been investigated as well, with the same aging times and storage conditions. The extraction procedure was conducted as reported in (Oasmaa et al., 2003). In brief, 400 g of water were added to 5 g of BCO in a Erlenmeyer flask so to separate the water insoluble fraction rich in lignin. The water-insoluble fraction was removed by filtration and further extracted with dichloromethane (i.e., CH_2_Cl_2_) so to obtain Low Molecular Mass (LMM, CH_2_Cl_2_-soluble part, about 400 Da) and High Molecular Mass (HMM, CH_2_Cl_2_-insoluble part, 1050 Da) lignin fractions. Both fractions were evaporated at 40°C overnight to remove any residue of CH_2_Cl_2_.

### 2.2 Neutron Scattering

In a neutron scattering measurement the experimentally determined quantity is the double differential scattering cross-section, $\frac{\partial^{2}\sigma }{\partial \Omega \partial E}$ , the probability that a neutron is scattered within the solid angle Ω+dΩ exchanging an energy E $<E_{i}$ -E_*f*_ < E + dE with the sample, E_*f*_ and E_*i*_ being the neutron final and initial energy, respectively.

Because of the way the neutrons are scattered by the nuclei in the sample, the scattering can be decomposed in the sum of two contributions: the coherent component, ${\left[\frac{\partial^{2}\sigma }{\partial \Omega \partial E}\right]}_{coh}$ , which yields information on the relative positions and motions of the atoms in the sample, and an incoherent contribution, ${\left[\frac{\partial^{2}\sigma }{\partial \Omega \partial E}\right]}_{inc}$ , which does not contain any structural information and only depends by total amount of the scatterers, yielding information on the single-particle dynamics:

$$\frac{\partial^{2}\sigma }{\partial \Omega \partial E}={\left[\frac{\partial^{2}\sigma }{\partial \Omega \partial E}\right]}_{coh}+{\left[\frac{\partial^{2}\sigma }{\partial \Omega \partial E}\right]}_{inc}$$ (1)

Neutron scattering probes the structure and dynamics at the nanoscale. The distances probed are determined by the inverse of the exchanged wavevector, **Q** = **k**_*i*_ − **k**_*f*_, where **k**_*i*_ and **k**_*f*_ are the initial and final wavevector of the scattered neutron. For isotropic samples the differential scattering cross section only depends on the modulus of *Q* = |**Q**|. In general, *Q* is a function of the scattering angle, *θ*, and the energies, *E*_*i*_ and *E*, *Q*(*θ*, *E*_*i*_, *E*); however, for small values of *E*, it is a function of the scattering angle and the wavelength of the incoming neutrons, *λ*, only:

$$Q=\frac{4\pi }{\lambda }\sin\left(\frac{\theta }{2}\right)$$ (2)

The microstructure of the BCO was investigated using SANS, for *Q* values in the range from 3 × 10^–3^ Å^−1^ to ≈0.6 Å^−1^, corresponding to length-scales approximately from 1 to 500 nm. In this range, the overwhelming contribution to the scattering is coherent and the incoherent component is just a *Q*-independent background.

The dynamics of the bio-oils was studied using QENS measurements for *Q* values 0.5 Å^−1^ ≤ Q ≤ 2.0 Å^−1^. In this *Q* range, to a first approximation, the scattering signal originates from the incoherent dynamics of the hydrogen atoms.

### 2.2.1 SANS

SANS is a static scattering technique which measures the scattered intensity without any analysis of the exchanged energy. Thus, it gives access to the differential scattering cross section:

$$\frac{\partial \sigma }{\partial \Omega }\approx \int_{-\infty }^{\infty }\left[\frac{\partial^{2}\sigma }{\partial \Omega \partial E}\right]dE$$ (3)

where the integration over the whole energy range is always approximated because of experimental constraints. Since in SANS the probed length-scales contain a relative large number of atoms, the scattering power can be expressed in terms of an average scattering length density:

$$\rho_{p}=\frac{\sum_{i}b_{i}^{coh}}{V_{p}}$$ (4)

where $b_{i}^{coh}$ is the coherent scattering length density of the i-th atom contained in the scattering object of volume *V*_*p*_.

Within certain approximations, the SANS intensity produced by a collection of uniform scatterers dispersed in a continuous medium (i.e., usually the dispersing medium or “solvent”) can be expressed as:

$$I\left(Q\right)=n_{p}{\left(\rho_{p}-\rho_{s}\right)}^{2}V_{p}^{2}P\left(Q\right)S^{SANS}\left(Q\right)+bkg$$ (5)

where *I*(*Q*) is the scattering intensity (i.e., the differential scattering cross section normalized by the scattering volume), *n*_*p*_ is the number density of the particles, $\left(\rho_{p}-\rho_{s}\right)$ represents the contrast between the scattering length density of the scattering object *ρ*_*p*_ and the one of the continuous medium *ρ*_*s*_, *V*_*p*_ is the volume of the scattering object, *P*(*Q*) is the form factor describing the shape of the scattering particles, *S* ^*SANS*^(*Q*) is the structure factor representing the Fourier transform of the relative position of the scatterers and *bkg* is the incoherent background.

The BCO samples investigated in this article can be considered as lignin dispersions in a mixture of water and organic molecules (that we have referred to as “solvent” in the previous section). Several structural models have been already screened for BCOs showing that the structure of the system in the liquid state can be better described by polydisperse clusters with a certain fractal nature originated by the aggregation of smaller lignin spherical units (Fratini et al., 2006). This approach is based on the work of Liu et al. (1995) where they followed the agglomeration of asphaltene primary units in liquid dispersions as a function of the asphaltene volume fraction and temperature. In the present case, considering aggregates consisting of *S* elementary spherical particles with radius, *R*_1_, fractal dimension, *D*_*f*_, degree of polydispersity on the average cluster size, *ν*, and assuming a continuous distribution of cluster size or small enough unit particle, the integral form of the scattering Eq. 5 reduces to:

$$I\left(Q\right)=\frac{{\left(\rho_{p}-\rho_{s}\right)}^{2}\phi V_{u}S}{\Gamma \left(2-\pi \right)}[F\left(3-\nu ,Q\xi \right){\left[1+Q^{2}\xi^{2}\right]}^{-D_{f}\left(3-\nu \right)/2}+G\left(2-\nu ,Q\xi \right){\left[\frac{Q\xi }{h}\right]}^{D_{f}}]+\frac{C_{P}}{Q^{4}}+bkg$$ (6)

where *ϕ* and *V*_*u*_ are, respectively the volume fraction and molecular volume of the base units; *ξ* is the correlation length of the fractal object defined as $\xi =hR_{1}S^{1/D_{f}}$ with $h=\sqrt{\frac{D_{f}\left(D_{f}+1\right)}{6}}$ . The functions *F* (*a*, *x*) and *G* (*a*, *x*) have the form:

F(a,x)=Γ(a)−Γ(a,u) (7)

and:

$$G\left(a,x\right)=\sin\left[\frac{\left(D_{f}-1\right)\pi }{2}\right]\frac{\Gamma \left(a,{\left(\frac{Q\xi }{h}\right)}^{D_{f}}\right)}{D_{f}-1}$$ (8)

with:

$$u={\left[\frac{h^{2}\left(1+Q^{2}\xi^{2}\right)}{Q^{2}\xi^{2}}\right]}^{D_{f}/2}$$ (9)

*x* = *Qξ* and Γ(*a*) and Γ(*a*, *b*) are the Gamma and incomplete Gamma function, respectively.

**FIGURE 1:**
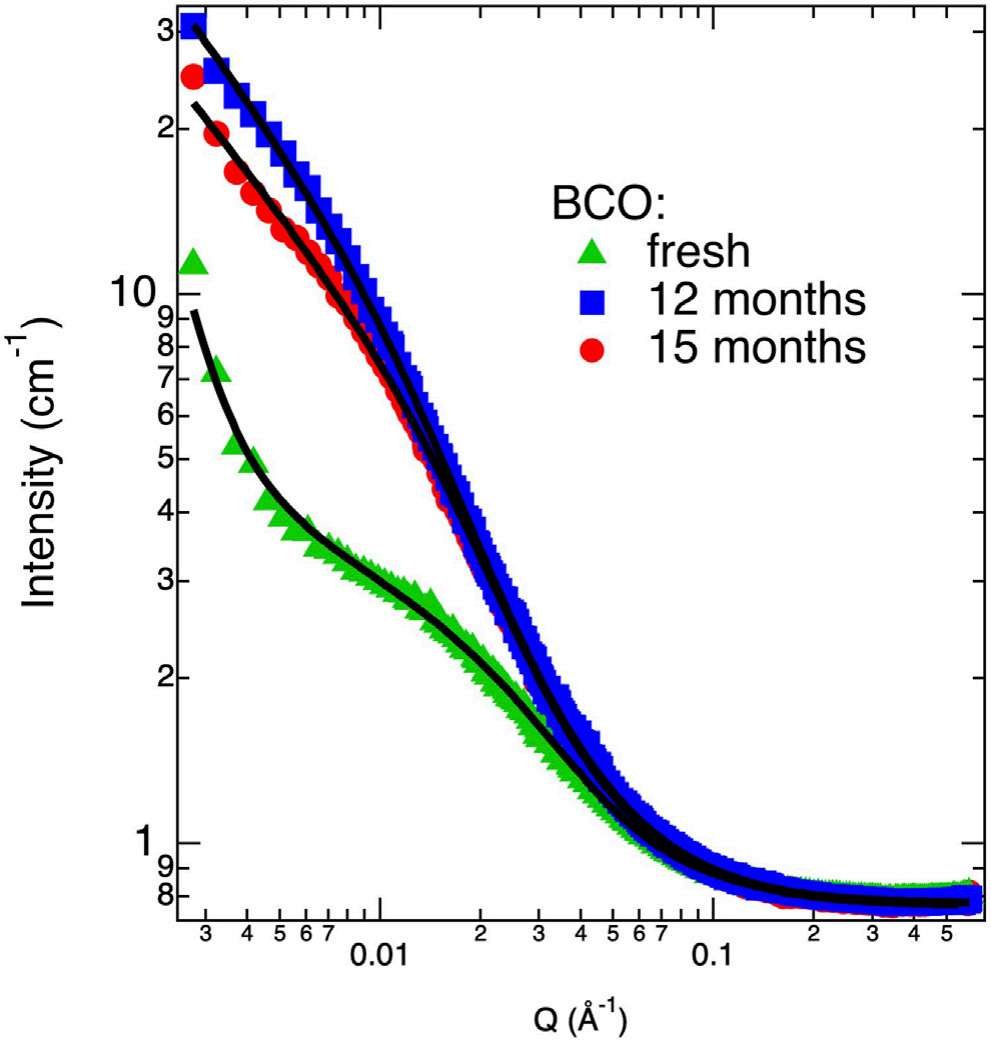
Scattering intensity profiles (markers) and correspondent best fits (lines) according to Eq. 6 for some of investigated BCO samples.

An additional Porod’s term with amplitude *C*_*P*_ and *Q* ^−4^ dependence is also included to take into account the low *Q* intensity increase present in the “fresh” sample (see Figure 1) and indicating the presence of aggregates at the upper limit of the dimensional range spanned by the SANS experiment (i.e., 2*π*/*Q*_min_).

Eq. 6 has been used in this work with *ϕ*, *S*, *D*_*f*_ and *ν* as adjustable parameters while R_1_ was kept fixed at 2.7 Å (Fratini et al., 2006). This model assumes the unit particles as monodisperse, a condition typical of the chemical nature of BCOs. The contrast between the lignin aggregates and continuous medium, (*ρ*_*p*_ − *ρ*_*s*_) can be calculated by the mass fraction of the scattering objects and the elemental analysis of the bio-oil and lignin as already described in (Fratini et al., 2006) using the composition and density values reported in Table 1.

### 2.2.2 QENS

Within the approximation that the overwhelming contribution to the neutron scattering originates from the incoherent scattering of the hydrogen atoms, the double differential scattering cross section for the QENS measurement is:

$$\frac{\partial^{2}\sigma }{\partial \Omega \partial E}\left(\theta ,E\right)=\hbar \frac{k_{f}}{k_{i}}N_{H}\frac{\sigma_{H}^{inc}}{4\pi }S_{H}^{self}\left(Q,E\right)⊗R\left(Q,E\right)$$ (10)

where N_*H*_ is the number of hydrogen atoms in the system, $\sigma_{H}^{inc}$ = 80.27 b is the incoherent scattering cross section of the hydrogen atoms, and *R*(*Q*, *E*) is the instrumental resolution function. $S_{H}^{self}\left(Q,E\right)$ is the single particle, i.e., self-dynamic structure factor of hydrogen atoms in the system:

$$\begin{array}{ccc} \hbar S_{H}^{self}\left(Q,E\right) & = & \int_{0}^{\infty }\left\langle \exp\left\{-iQ\cdot \left[r_{H}\left(t\right)-r_{H}\left(0\right)\right]\right\}\right\rangle \exp\left\{-i\frac{E\;t}{\hbar }\right\}dt=\\ & & \int_{0}^{\infty }F_{H}^{self}\left(Q,t\right)\exp\left\{-iEt_{\hbar }\right\}dt \end{array}$$ (11)

where $F_{H}^{self}\left(Q,t\right)$ is the intermediate scattering function, ISF, and ⟨…⟩ indicates an ensemble average.

In particular, Elastic Neutron Scattering (ENS) measurements determine the value of the differential scattering cross-section for *E* = 0, within the instrumental resolution:

$$\begin{array}{ccc} \frac{\partial^{2}\sigma }{\partial \Omega \partial E}\left(\theta ,E\approx 0\right) & = & \hbar \frac{k_{f}}{k_{i}}N_{H}\frac{\sigma_{H}^{inc}}{4\pi }\int_{-\infty }^{\infty }S_{H}^{self}\left(Q,E\right)\times R\left(Q,E\right)dE=\\ & & \hbar \frac{k_{f}}{k_{i}}N_{H}\frac{\sigma_{H}^{inc}}{4\pi }\int_{0}^{\infty }F_{H}^{self}\left(Q,t\right)\times R^{t}\left(Q,t\right)dt \end{array}$$ (12)

*R* ^*t*^(*Q*, *t*) being the time Fourier transform of the instrumental resolution function (Fratini et al., 2013).

The position of the H atoms can be decomposed as the sum of the position of a reference point, **r**_*R*_, such as the center of mass of the molecule, the relative position with respect to the reference point, **b**, and the vector defining the vibrational displacement from the bond equilibrium position, **u**:

$$r_{H}=r_{R}+b+u$$ (13)

Within the decoupling approximation, the motions described by these three vectors, namely translational, reorientational and conformational, and vibrational, are statistically independent, hence:

$$\begin{array}{ccc} \langle \exp\left\{-iQ\cdot \left[r_{H}\left(t\right)-r_{H}\left(0\right)\right]\right\}\rangle \approx \langle \exp\left\{-iQ\cdot \left[r_{R}\left(t\right)-r_{R}\left(0\right)\right]\right\}\rangle \times & & \\ \langle \exp\left\{-iQ\cdot \left[b\left(t\right)-b\left(0\right)\right]\right\}\rangle \times \langle \exp\left\{-iQ\cdot \left[u\left(t\right)-u\left(0\right)\right]\right\}\rangle & & \end{array}$$ (14)

Bond vibrational motions are too fast to fall within the instrumental energy window and only cause a reduction of the total scattering intensity in the QENS window. In terms of its ISF, $F_{rc}^{self}\left(Q,t\right)$ , or its dynamic structure factor, $S_{rc}^{self}\left(Q,E\right)$ , the reorientational and conformational dynamics can be expressed as the sum of products of *Q* dependent and time dependent factors:

$$\begin{array}{ccc} F_{rc}^{self}\left(Q,t\right) & = & A_{0}\left(Q\right)+\underset{l}{\sum }A_{l}\left(Q\right)\exp\left\{-\Gamma_{l}t\right\} \end{array}$$ (15)

$$\begin{array}{ccc} S_{rc}^{self}\left(Q,E\right) & = & A_{0}\left(Q\right)\delta \left(E\right)+\underset{l}{\sum }A_{l}\left(Q\right)Lor\left(\Gamma_{l}\right) \end{array}$$ (16)

where Lor(Γ_*l*_) represents a Lorentzian function with a full width half maximum equal to Γ_*l*_. The reorientational and conformational dynamics is characterized by a time independent term, which in the energy domain results in the presence of a delta function, i.e., a resolution limited feature centered at *E* = 0. The *Q* dependence of this contribution, *A*_0_(*Q*), is referred to as the Elastic Incoherent Structure Factor (EISF) and represents the spatial Fourier transform of the volume explored by the hydrogen atoms during their motion, with respect to the reference point.

Finally, as far as the dynamics of **r**_*R*_ is concerned, when the point of reference represents the center of mass of a molecule in the liquid phase (a common choice to model the data in liquids), the corresponding dynamics represents the translational motion of the molecule. Models corresponding for example to Fickian, jump, or confined diffusion have been developed, generally characterized by a *Q* dependence of the characteristic relaxation time.

In water and other liquids, the accuracy of the decoupling approximation between the translational and reorientational and conformational motions is limited to several percent and it is hard to determine *a priori*, therefore, even if employed very commonly, it represents a major limitation to the accurate interpretation of QENS results (Faraone et al., 2003).

### 2.3 Instrumentation

SANS measurements were performed on D22 at the Institute Laue Langevin (ILL), Grenoble, France. The incoming neutron wavelengths was set to 6 Å with a Δ*λ*/*λ* ≈ 10*%*. Three sample to detector distances were employed to cover a *Q* range from 3 × 10^–3^ Å^−1^ to about 0.6 Å^−1^. Data were corrected for the dark counts intensity and the contribution from the empty containers and converted to 1D absolute intensity using standard routines at ILL. All the SANS measurements were carried out using Hellma quartz cells of 1 mm path length at 20.0°C ± 0.1°C.

Elastic scan measurements were performed on the High Flux Backscattering Spectrometer (HFBS) at National Institute of Standards and Technology (NIST) Center for Neutron Research (NCNR), in Gaithersburg, MD, United States. In this operation mode both the initial and final energy of the neutrons detected is determined by Bragg reflection of the neutrons by the Si(111) crystals of both the monochromator and the analyzers. To ensure minimal wavelength (i.e., energy) spread of the detected neutrons, the analyzers are arranged in such a way that only backscattered neutrons of the required energy satisfy the Bragg condition and reach the detectors. The corresponding instrumental resolution is of ≈0.8 *μ*eV. A set of detectors records the intensity of neutrons for 16 *Q* values. The elastic scattered intensity is recorded as the sample temperature is changed using a cold cycle refrigerator with a temperature accuracy of ≈0.1 K. The temperature scan rate was 1/60 K/s. Samples were spread in thin annular sheets of ≈0.5 mm thickness, to minimize multiple scattering, and contained in aluminum cans sealed with indium.

QENS Time-of-Flight measurements were performed on the cold disk chopper IN5 at ILL. An incoming wavelength of 5 Å was selected. The instrumental resolution function could be well reproduced by a Gaussian function with a Full Width at Half Maximum varying from ≈82 *μ*eV to ≈88 *μ*eV depending on the scattering angle. Data were converted to sets of 16 constant *Q* spectra in the range from 0.5 Å^−1^ to 2.0 Å^−1^ using standard routines available in LAMPS.

Both ENS and QENS data were analyzed using DAVE (Azuah et al., 2009).

## 3 Results and Discussion

### 3.1 SANS Results

Figure 1 shows the SANS curves of some of the BCO samples investigated in this study. As it can be clearly depicted, the scattering signal increases passing from the “fresh” to the “12 months” sample. The “15 months” sample shows a small decrease in the scattered intensity with respect of the “12 months” sample, most reasonably due to a partial sedimentation of larger aggregates. Fitting these curves with Eq. 6 allows for a quantitative picture of the evolution of BCO structure at the nanoscale. Table 2 lists the results of the best fits done according to Eq. 6. In particular, all the samples can be modeled using the structure factor associated to a fractal assembly of spherical lignin units of about 2.7 Å. In the case of the “fresh” sample an extra term accounting for the Porod’s contribution from larger aggregates is also included (i.e., the scattering coming from the surface of the aggregate). This contribution does not provide any benefit to the final fits in all other samples probably because this fraction of larger objects precipitates out of the sample at longer times or it is covered by the scattering of the growing fractal assembly. The aggregation parameter, S, (i.e., the average number of units present in the fractal assembly) increases from 189 for the fresh sample to 886 after 12 months of aging. This value decreases to 626 after 15 months of aging, consistently with the hypothesis of sedimentation of part of the larger aggregates. The fractal dimension associated to the clusters are slightly increasing with the aging time, but they are in all cases around 1.9. More important, the polydispersity parameter *ν* shows a significant increase as a function of aging time from 1.2 to 1.4. Values of *S* and *D*_*f*_ are greater than the ones reported in the literature on a batch of BCO produced by VTT starting from different biomass and production plant (Fratini et al., 2006) thus indicating that the lignin aggregates formed in the present case are more clustered and have a denser structure. The index *ν* gives information on the degree of polydispersity associated to the cluster size and more important reveals the mechanisms behind cluster formation. Smaller *ν* are found in the case of broader cluster size distribution with values around 1.4 typical for reaction limited aggregation (RLA) and around 1.9 in the case of diffusion limited mechanism (DLA) (Chen et al., 1992). Value of *ν* obtained in the present investigation are slightly lower than 1.4 showing that the clustering mechanisms is dominated by a RLA process. The high value of the incoherent background are in line with the high hydrogen content evidenced by the CHNO analysis.

**TABLE 2:** Best fitting parameters obtained from SANS analysis by using Eq. 6 with R_1_ = 2.7 Å.

	Fresh	12 months	15 months
*S*	189 ± 2	886 ± 14	626 ± 8
*D*_*f*_	1.87 ± 0.01	1.92 ± 0.03	1.94 ± 0.01
*ν*	1.19 ± 0.01	1.27 ± 0.01	1.39 ± 0.01
*C*_*P*_ (⋅10^–10^)	2.77 ± 0.15	—	—
*bkg*	0.773 ± 0.001	0.778 ± 0.002	0.769 ± 0.002

### 3.2 ENS Results

Within the Guassian approximation the single particle ISF is related to the mean squared displacement (*msd*) of the hydrogen atoms.

$$S_{H}^{self}\left(Q,E\right)=F\left\{\exp\left\{-\frac{1}{6}Q^{2}\left\langle {\left[r\left(t\right)-r\left(0\right)\right]}^{2}\right\rangle \right\}\right\}$$ (17)

where F{} represents a Fourier transform operation.

Within some approximations, from Eqs 12, 17, the *msd* over the timescale determined by the energy resolution of the spectrometer, ≈ 1 ns in the HFBS case, can be derived as a linear fit to the data:

$$-3\ln\left(\frac{\frac{\partial^{2}\sigma }{\partial \Omega \partial E}\left(Q,E\approx 0,T\right)}{\frac{\partial^{2}\sigma }{\partial \Omega \partial E}\left(Q,E\approx 0,T_{0}\right)}\right)=I_{0}+msd\left(T\right)\times Q^{2}$$ (18)

where *T*_0_ is the lowest temperature measured, for which it is assumed that no significant dynamics is observable. *I*_0_ is the intercept of the linear fit in *Q* ^2^, accounting for experimental errors as well as for the presence of non Gaussian contributions.

**FIGURE 2:**
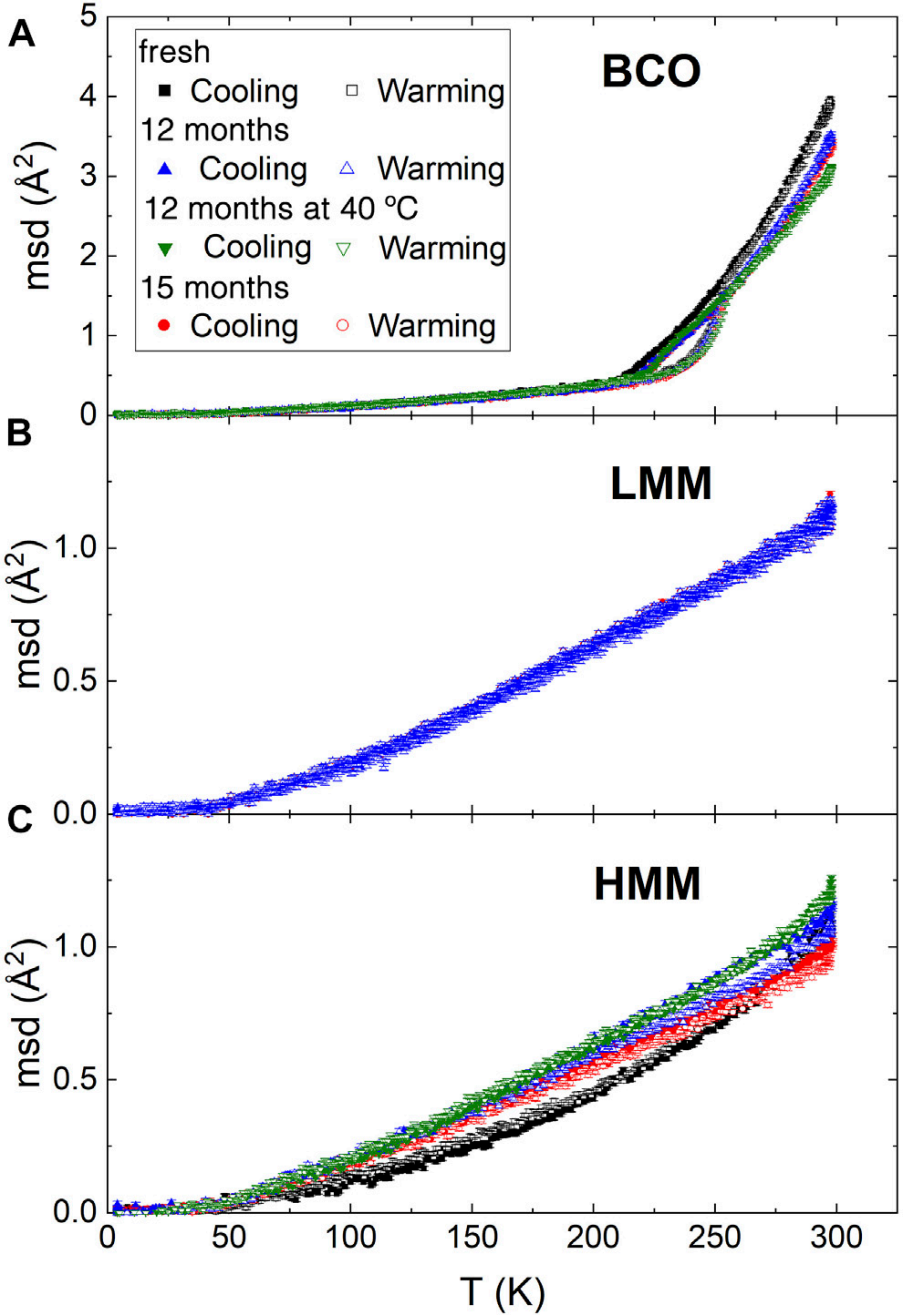
Temperature dependence of the mean squared displacement over a timescale of ≈1 ns, obtained from the elastic neutron scattering measurements on HFBS. Samples at four aging stages were tested as indicated in the legend of plot **(A)** [The legend applies to plot **(B,C)** as well]. Measurements for each sample were performed first cooling and then heating it back again to 300 K. Plot **(A,B,C)** report the results for BCO, low and high molecular mass lignin fractions, respectively.

Figure 2 reports the temperature dependence of the *msd* of the hydrogen atoms for the present investigation. Although for BCO it is much less pronounced, all the samples display an upturn of the curves at ≈ 50 K which is associated to the activation of methyl groups rotation (Senses et al., 2018). The *msd* of the BCO samples is characterized by a hysteresis loop in the range from 200 to 250 K. Interestingly, BCO can be cooled without displaying a sudden drop in the *msd* which is the hallmark of freezing. A rather sharp change in the slope of the curve is observed at ≈ 220 K, which can be associated to a liquid to glass transition. This temperature is not far from the much discussed dynamic transition temperature in proteins (Doster et al., 1989) and other water hydrated systems (Tavagnacco et al., 2019), which is believed to be intimately related to the dynamics of interfacial water (Chen et al., 2006). This result suggests that lowering the storing temperature below this transition threshold might be a way to suppress the reactivity of the BCO. On the other hand in the hysteresis loop, on warming, a fast increase of the *msd* just above 225 K is followed by a drastic change of slope at 250 K, overlapping with the cooling data. This unusual pattern of the *msd* curves indicates the frustration of crystallization of the liquids because of confinement and interfacial interactions. As a comparison, water confined within nanoporous matrices with pores of ≈28 Å and ≈24 Å display a depression of the freezing point of more than 50°C and 70°C, respectively; whereas for pores of ≈20 Å diameter, no freezing signature can be observed even with differential scanning calorimetry (Yoshida et al., 2008).

Above 250 K, in the liquid phase, the *msd* of BCO decreases with aging. However, this reduction in mobility is not monotonic as indicated by the fact that the “12 months” and “15 months” samples show similar values of the *msd*. Remarkably, the “12 months at 40°C” sample displays slightly smaller *msd* values which could be a consequence of the greater reactivity of the sample stored at 40°C leading to an increased amount of water, as well as other liquids, in the confined and interfacial state. The increase in size of the carbonaceous aggregates supports this picture.

As a comparison, the behavior of the pyrolytic lignin fractions is quite different. The absolute value of the *msd*, even at the highest temperature investigated of 300 K, is of about 1 Å^2^; much lower than the one encountered for BCO. This is not surprising considering that lignin is a solid phase and therefore no long distance dynamics is expected. Moreover, beyond the already discussed upturn of the *msd* at 50 K, no other significant feature is present in the whole temperature range. The warming and cooling scans do not show any hysteresis consistently with the absence of phase changes in these matrices. Although the low molecular weight lignin display no dependence on aging, slight changes are observed in the high molecular weight case. In these samples, the mobility of the hydrogen atoms increases with aging. The chemical reactions taking place induce a rearrangement of the molecular and chemical structure of the lignin which allows for a slightly increased molecular mobility. It could be speculated, although no direct evidence can be provided, that the same changes take place in the lignin components of the BCO during aging, as a result of similar chemical reactions and structural rearrangements. The increase in size of the aggregates observed by SANS is consistent with this scenario. Interestingly, around room temperature, which is the most relevant range for the applications of these products, the increase of the *msd* is much larger for the sample aged at 40°C with respect to the others, confirming the higher reactivity of this sample.

### 3.3 QENS Results

**FIGURE 3:**
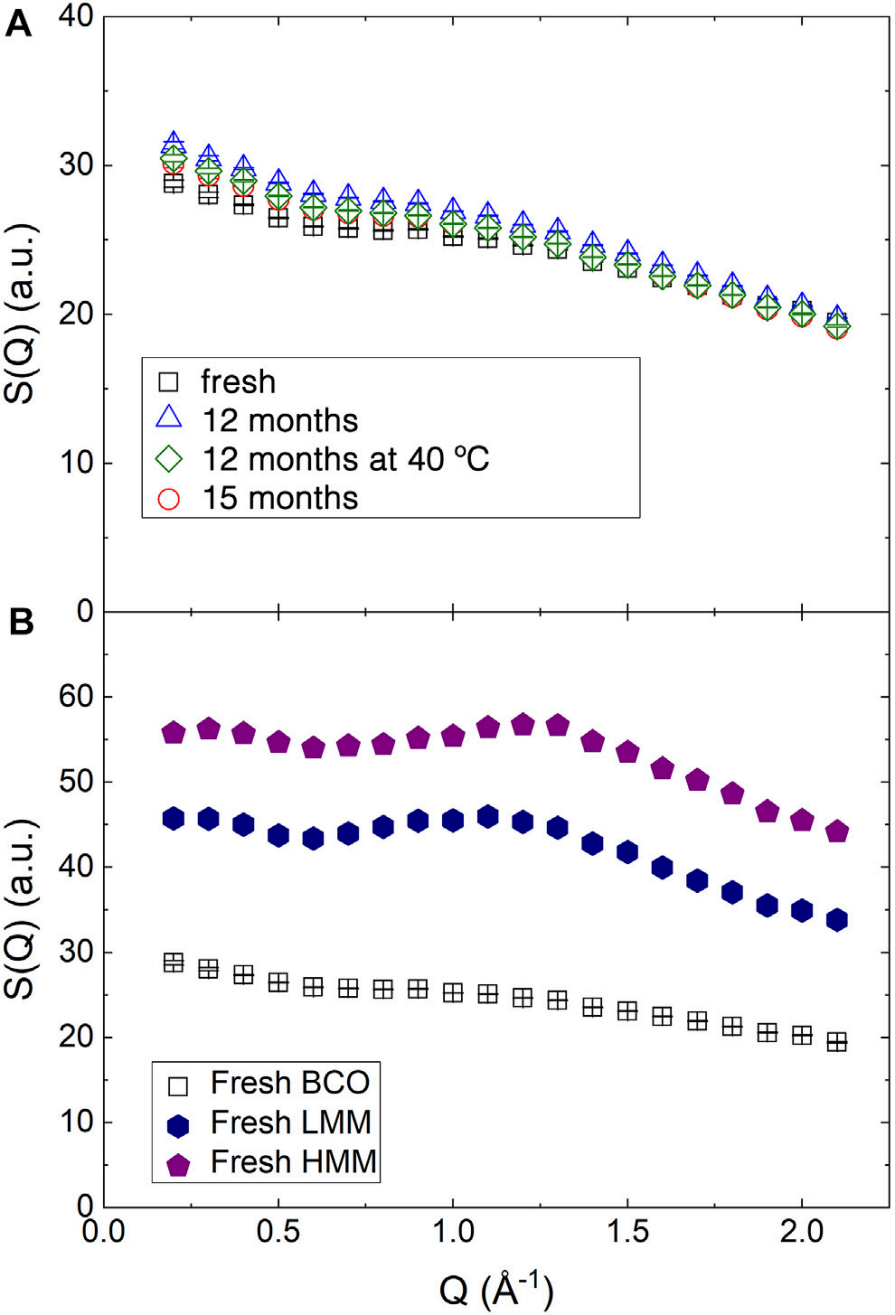
Structure factor as obtained integrating the QENS spectra collected on IN5 over *E* (see Eq. 3). **(A)** BCO at four aging stages. **(B)** The structure factor of fresh BCO is compared to the structure factor of the fresh low and high molecular mass lignin fractions.

The structure of the samples at molecular length scales was investigated by integrating the QENS spectra over the available energy range to obtain an approximation of the structure factor, as indicated in Eq. 3. The obtained results are reported in Figure 3. For BCO, as shown in panel (a) the slight decrease of *S*(*Q*) up to ≈0.70 Å^−1^ is likely due to the high *Q* tail of the SANS pattern and indicates the presence of not negligible coherent neutron scattering contributions. The further decrease at higher *Q* values is instead related to the limited energy range covered in the integration. Small differences can be observed among the samples differing for their aging even if the trends are not monotonic and difficult to be interpreted.

Figure 3B reports the structure factor for the fresh lignin fractions. The broad peak observed in the middle of the reported *Q* range is similar to the one observed in amorphous cellulose using X-ray diffraction (Park et al., 2010) and Kraft lignin (Goudarzi et al., 2014). The slight increase of the peak position in the HMM sample indicates a more compact structure of the low molecular weight lignin.

The presence of structural features in the samples indicates that the interpretation of the QENS results cannot neglect the presence of residual coherent signal beside the single particle dynamics of the hydrogen atoms. Moreover, the inherent inhomogeneity of the sample and the complexity introduced by confinement prevent from developing a detailed model of the microscopic motion of the hydrogen atoms. Therefore, an empirical approach is employed to gain information on the general geometry and timescales of the motions.

**FIGURE 4:**
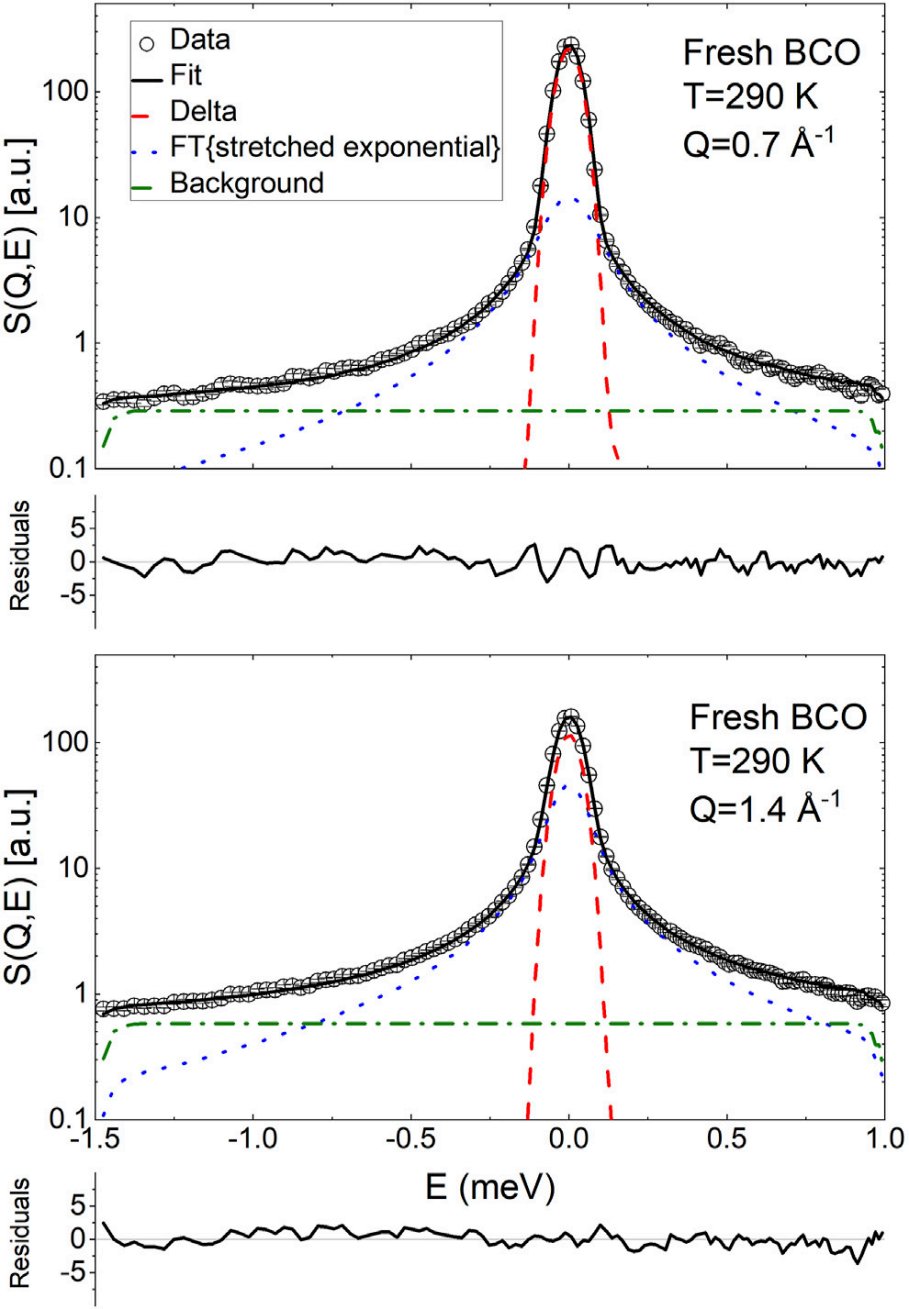
Exemplary fit of the QENS spectra of fresh BCO using Eq. 20 at two *Q* values. The normalized residuals ( $\frac{\exp\;data-fit}{error\;bar}$ ) are plotted to provide an indication of the quality of the fit.

**FIGURE 5:**
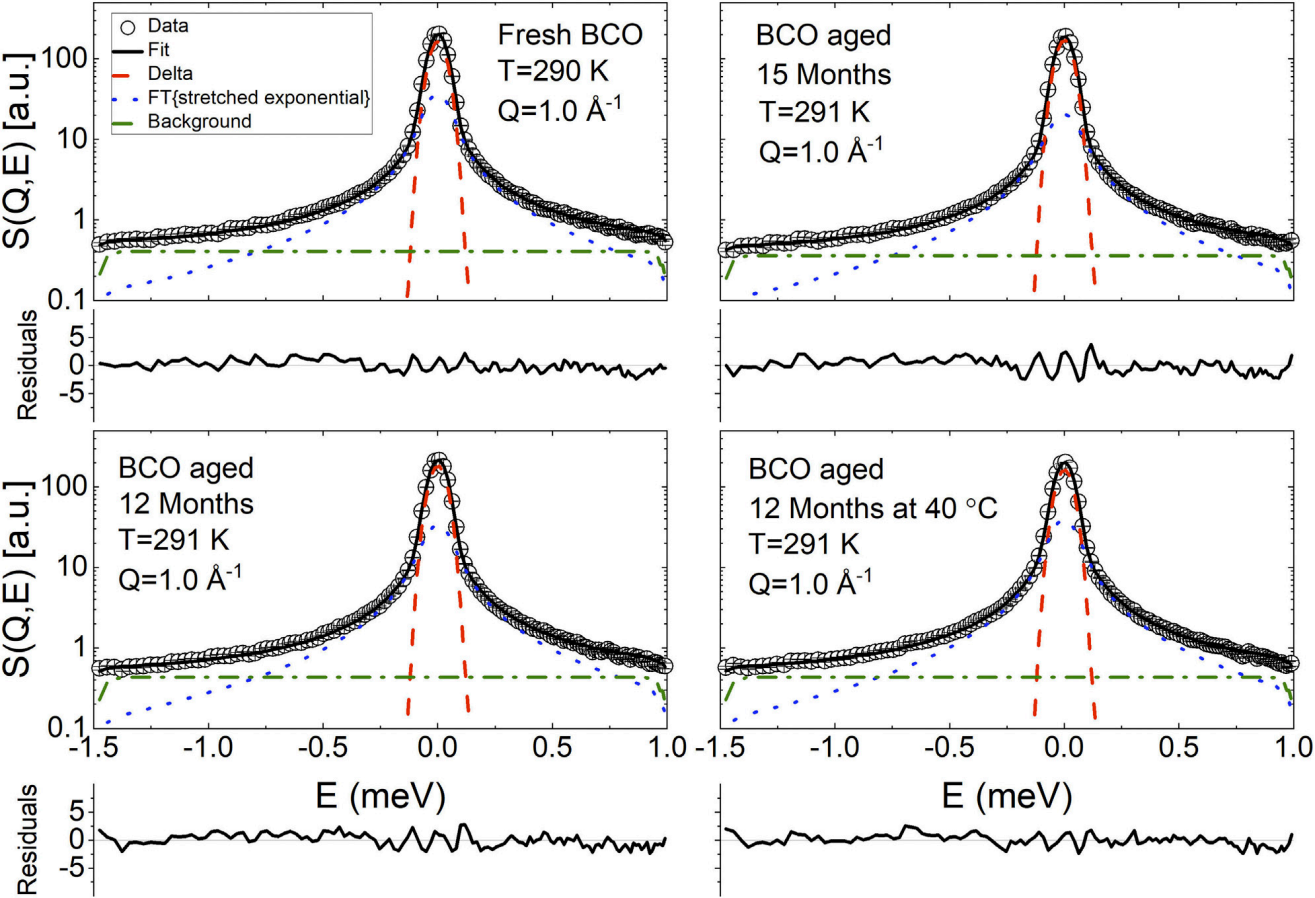
Exemplary fit of the QENS spectra of BCO at four aging stages using Eq. 20. The normalized residuals ( $\frac{\exp\;data-fit}{error\;bar}$ ) are plotted to provide an indication of the quality of the fit.

Figures 4, 5 display the QENS spectra at two *Q* values (0.7 and 1.4 Å) for fresh BCO and for the aging stages of BCO investigated at *Q* = 1 Å^−1^, respectively. The spectra are characterized by the presence of a resolution limited component, from now on referred as delta. Therefore, the ISF of the single particle dynamics of the hydrogen atoms has been modeled as the sum of stretched exponential decay and a constant background:

$$S_{H}^{self}\left(Q,E\right)=A^{\delta }\left(Q\right)+\left[1-A^{\delta }\left(Q\right)\right]F\left\{\exp\left\{-{\left[\frac{t}{\tau \left(Q\right)}\right]}^{\beta \left(Q\right)}\right\}\right\}$$ (19)

The time independent term, *A* ^*δ*^(*Q*), accounts for the presence of immobile^1^ hydrogen atoms in the aggregates. Moreover, the presence of this time independent term can be originated by a rotational and reorientational dynamics of the hydrogen atoms in a molecule, when diffusion of the point of reference, i.e., the center of mass, is too slow to be appreciated by the spectrometer employed because of its limited resolution. The stretched exponential function is commonly employed as an empirical way to describe relaxation data that do not follow a simple Debye behavior. The case *β* = 1 coincides with an exponential relaxation, whereas values of *β* increasingly smaller than one indicate higher degrees of non-exponential behaviour. A stretched behavior of the ISF can originate either from a distribution of exponential relaxations with different characteristic times or from intrinsically non exponential relaxation processes (Colmenero et al., 1999).

Therefore, considering the molecular heterogeneity of the BCOs giving rise to a distribution of exponential relaxation times, the data have been analyzed using the following fitting function:

$$S\left(Q,E\right)=A\left(Q\right)\{A^{\delta }\left(Q\right)\delta \left(E\right)+\left[1-A^{\delta }\left(Q\right)\right]F\left\{\exp\left[-{\left[\frac{t}{\tau \left(Q\right)}\right]}^{\beta \left(Q\right)}\right]\right\}⊗R\left(Q,E\right)\}+bkg$$ (20)

where A(Q) represents the total spectral intensity, *δ*(E) is a Dirac delta function, and *bkg* is a background which accounts for fast dynamical processes outside the instrumental window as well as instrumental background contributions.

Besides Eq. 20, several other fitting equations have been employed. None of the models which did not contain an immobile contribution was able to describe the spectra satisfactorily or would yield nonphysical results. Using the sum of a delta function and a Lorentzian, i.e., forcing *β*(*Q*) = 1 in Eq. 20, also yielded unsatisfactory fits. Using the sum of a delta and two Lorentzian functions allowed to obtain good fitting; however, since the model of Eq. 20 has one less fitting parameter and the overall results of the models were the same, only the results of the fitting from Eq. 20 are presented. The reader is cautioned, however, to keep in mind that, given the complexity of the samples and the limited *Q*/*E* probed, the present results should be considered preliminary in the sense that, as more information on the samples are gathered from other measurements and investigations, a more refined and insightful fitting model might be developed.

**FIGURE 6:**
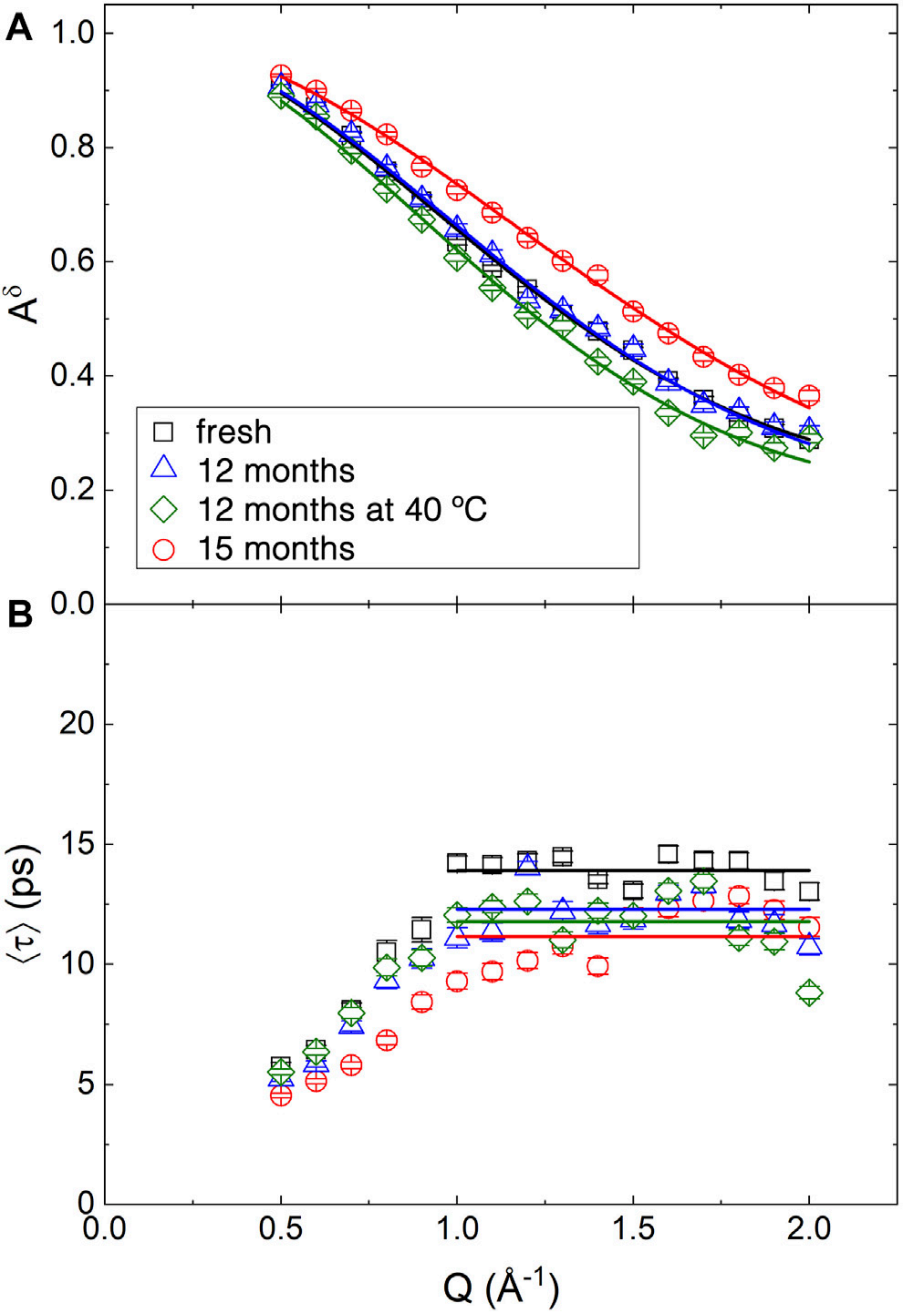
*Q* dependence of the best fitting parameters obtained from the QENS spectra of BCO at four aging stages using Eq. 20. **(A)** Continuous lines are the fitting according to Eq. 21. **(B)** Straight lines indicate the expected plateau value of ⟨*τ*⟩ for *Q* ≥1 Å^−1^.

Figure 6 reports the main results on the microscopic dynamics of the hydrogen atoms. Plot (a) shows the *Q* dependence of *A* ^*δ*^. As discussed before, the delta contribution to the spectra arises both from immobile hydrogen atoms (over all length scales) and from the reorientational and conformational dynamics of the hydrogen atoms with respect to a reference point immobile on the instrumental time scale. Therefore, *A* ^*δ*^(*Q*) results have been analyzed using the equation:

$$A^{\delta }\left(Q\right)=\left(1-A^{imm}\right)*\exp\left[-\frac{{\left(QR_{g}^{rc}\right)}^{2}}{3}\right]+A^{imm}$$ (21)

*A* ^imm^ represents the fraction of immobile atoms. The Gaussian term is analogue to the Guinier expression used to model the low-*Q* region of the SANS curves. Here it is used to extract a characteristic size of the region explored by the hydrogen atoms in their reorientational and conformational motion.

The stretched exponential function, often also referred to as the Kolrausch-Williams-Watts (KWW) function (Williams and Watts, 1970), can be considered as the results of the presence of a distribution of exponential relaxation. The first moment of the distribution defines the average relaxation time of the distribution, ⟨*τ*⟩ (Johnston, 2006):

$$\left\langle \tau \right\rangle =\frac{\tau }{\beta }\Gamma \left(\frac{1}{\beta }\right)$$ (22)

The *Q* dependence of ⟨*τ*⟩ is reported in Figure 6B. The increase of ⟨*τ*⟩ with *Q* for *Q* < 1.0 Å^−1^ is not physical. It is probably related to the strong correlation in the fitting between the various fitting parameters. Moreover, the presence of a coherent contribution at low *Q*, as observed in Figure 3 might introduce some artifacts. Above *Q* = 1 Å^−1^ the data fluctuate around an average value indicated by the horizontal lines in the figure. In fact, as indicated by Eq. 15, for a reorientational and conformational (*rc*) type of dynamics, the timescale of the motion should be time independent. In a liquid, because of the diffusive motion, a *Q* ^2^ dependence of the broadening should be observed. The absence of an appreciable diffusive contribution in the sample indicates that the long distance translational motion of the molecules in the liquid phase is too slow to be appreciated. Fresh BCO appears as a sticky substance and has a macroscopic viscosity 65 times larger than water (Chiaramonti et al., 2011); although the mobility at the atomic level might not scale with the viscosity, this occurrence explains the absence of a diffusive component in the spectra.

Table 3 reports the results of the fitting of *A* ^*δ*^(Q). The average value of ⟨*τ*⟩ as well as of the stretching exponent *β*, are also reported as $\bar{\left\langle \tau \right\rangle }$ and $\bar{\beta }$ . The behavior of the parameters with aging is not monotonic. The volume explored by the hydrogen atoms in fresh BCO has a characteristic size of ≈1.3 Å. This can be compared to the distance between the hydrogen atom and the center of mass in water, 0.98 Å, or the typical radius of methyl groups rotation. Hence, the motion is highly restricted to local rearrangements of the chemical bonds or roto-translational motion of water and the other hydrogenated liquids on the surface of the carbonaceous aggregates. Interestingly, the *R*_*g*_ obtained in the 15 months aged BCO is significantly while both the 12 months aged samples retain *R*_*g*_ values similar to the one of fresh BCO. The value of *A* ^imm^, of the order of 20%, is comparable to the volume fraction of the fractal aggregates observed in SANS. On the other hand, *A* ^imm^, displays with aging the opposite trend as the one observed in *R*_*g*_, with the smallest immobile fraction observed in the 15 months sample, and similar values recorded for the fresh and 12 months aged samples. However, these results points to the fact that as the chemical reactions take place in the BCO matrix with aging, an increasing number of molecules is comprising the carbonaceous aggregates or is bound and trapped within them; the remaining fractions of molecules being the ones with increased mobility.

**TABLE 3:** Fitting results of the Q dependence of the parameters extracted from the QENS spectra.

	Rg (Å)	A^imm^	$\bar{\left\langle \tau \right\rangle }$ (ps)	$\bar{\beta }$
BCO				
Fresh	1.31 ± 0.02	0.21 ± 0.02	13.9 ± 0.2	0.603 ± 0.003
12 months	1.26 ± 0.04	0.18 ± 0.02	12.3 ± 0.3	0.622 ± 0.004
12 months at 40°C	1.37 ± 0.03	0.18 ± 0.02	11.8 ± 0.4	0.638 ± 0.003
15 months	1.06 ± 0.02	0.16 ± 0.02	11.2 ± 0.4	0.625 ± 0.004
Lignin				
LMM	1.52 ± 0.05	0.869 ± 0.003	4.02 ± 0.06	1 ± 0
HMM	1.21 ± 0.06	0.833 ± 0.008	4.80 ± 0.13	1 ± 0

The time scale of the motion is of the order of 10 ps. This can be compared with the timescale of the microscopic mobility of water: in fact, water molecules are believed to diffuse in the liquid through a sequence of random jumps. The time spent by the water molecules in between jumps, the residence time, has been measured using QENS (Teixeira et al., 1985). Even if the QENS spectra of BCO do not demonstrate a diffusive like Q^2^ dependence of the quasielastic broadening, the residence time is akin to the timescale measured in the present experiment, because it is related to the broadening of the QENS spectra at high *Q*, in a *Q* independent region. The values found for BCO are comparable to the ones observed in bulk water at ≈ 250 K. This confirm the extreme confinement experienced by the liquid phases in BCO: in fact, it was proposed that interfacial water displays the same dynamics of bulk water at a temperature ≈20 K lower (Chen et al., 1995). Cured cement, also provides an interesting point of comparison, since it is characterized by a significant fraction of interfacial water which plays a major role in its continuous curing. Also in QENS data from cement, the *Q* dependence of interfacial water dynamics is observed to increasingly deviate from the expected diffusive behavior as curing goes on (Fratini et al., 2001). Moreover, as a comparison, in cement timescales of the order of 10 ps were observed in the dynamics of the interfacial water after few days of curing (Fratini et al., 2002). Once again we observe that the 15 months sample mobile hydrogen atoms display the slower dynamics with the 12 months aged samples approaching again the values of fresh BCO. The stretching exponent $\bar{\beta }$ indicates the presence of a broad distribution of relaxation time with a slight decrease of the non-exponential behaviour from the fresh to the BCO sample aged at 40°C.

**FIGURE 7:**
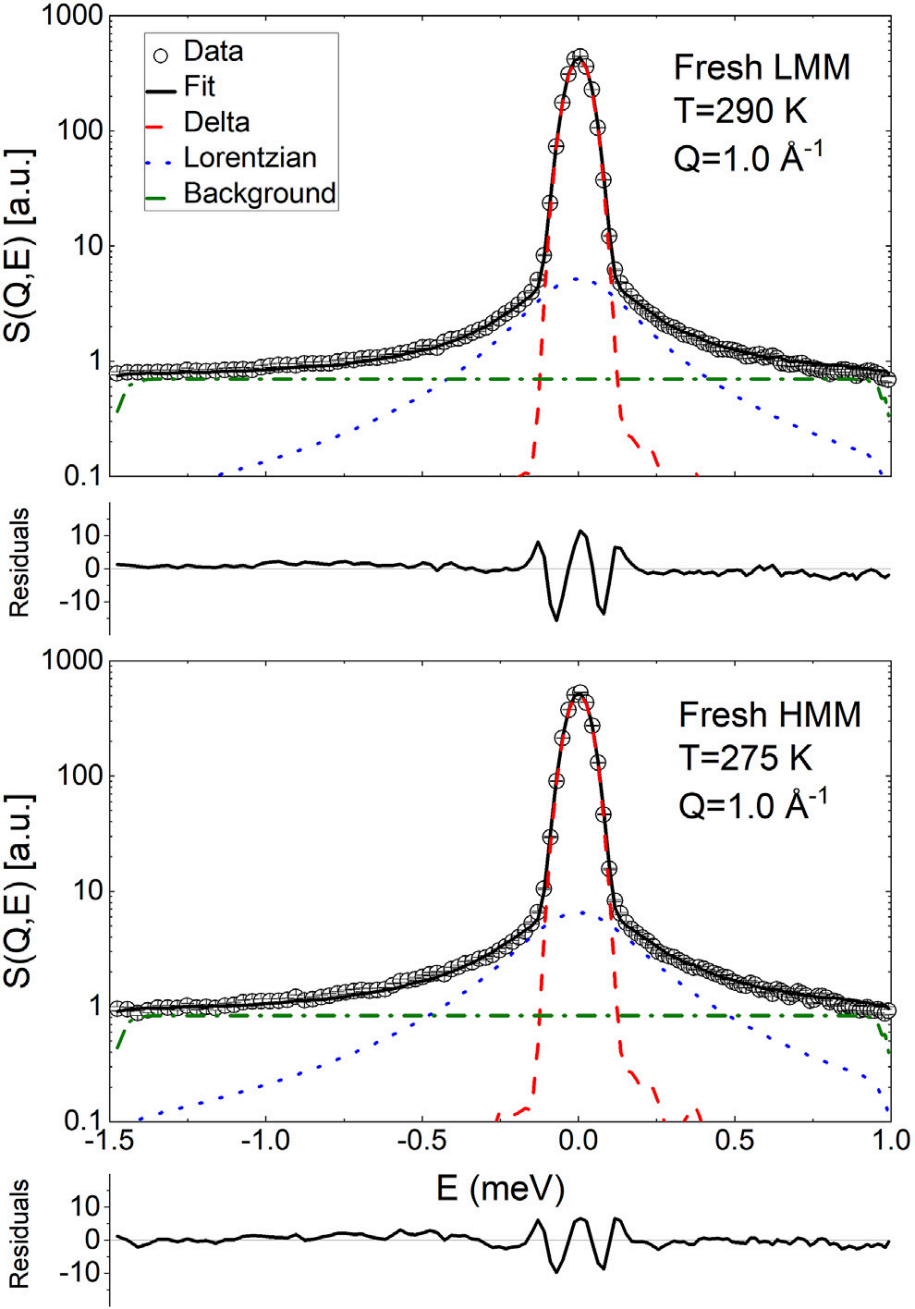
Exemplary fit of the QENS spectra of fresh low and high molecular mass pyrolytic lignin using Eq. 20. The normalized residuals ( $\frac{\exp\;data-fit}{error\;bar}$ ) are plotted to provide an indication of the quality of the fit.

QENS measurements were also performed on samples of fresh LMM and HMM lignin fractions. The data were analyzed according to Eq. 20; however, the obtained values of *β* were close to unity and therefore the parameter was fixed to 1. Hence, the QENS data were essentially fitted using the sum of a delta function and a Lorentzian broadening. Examples of the fitting are shown in Figure 7.

**FIGURE 8:**
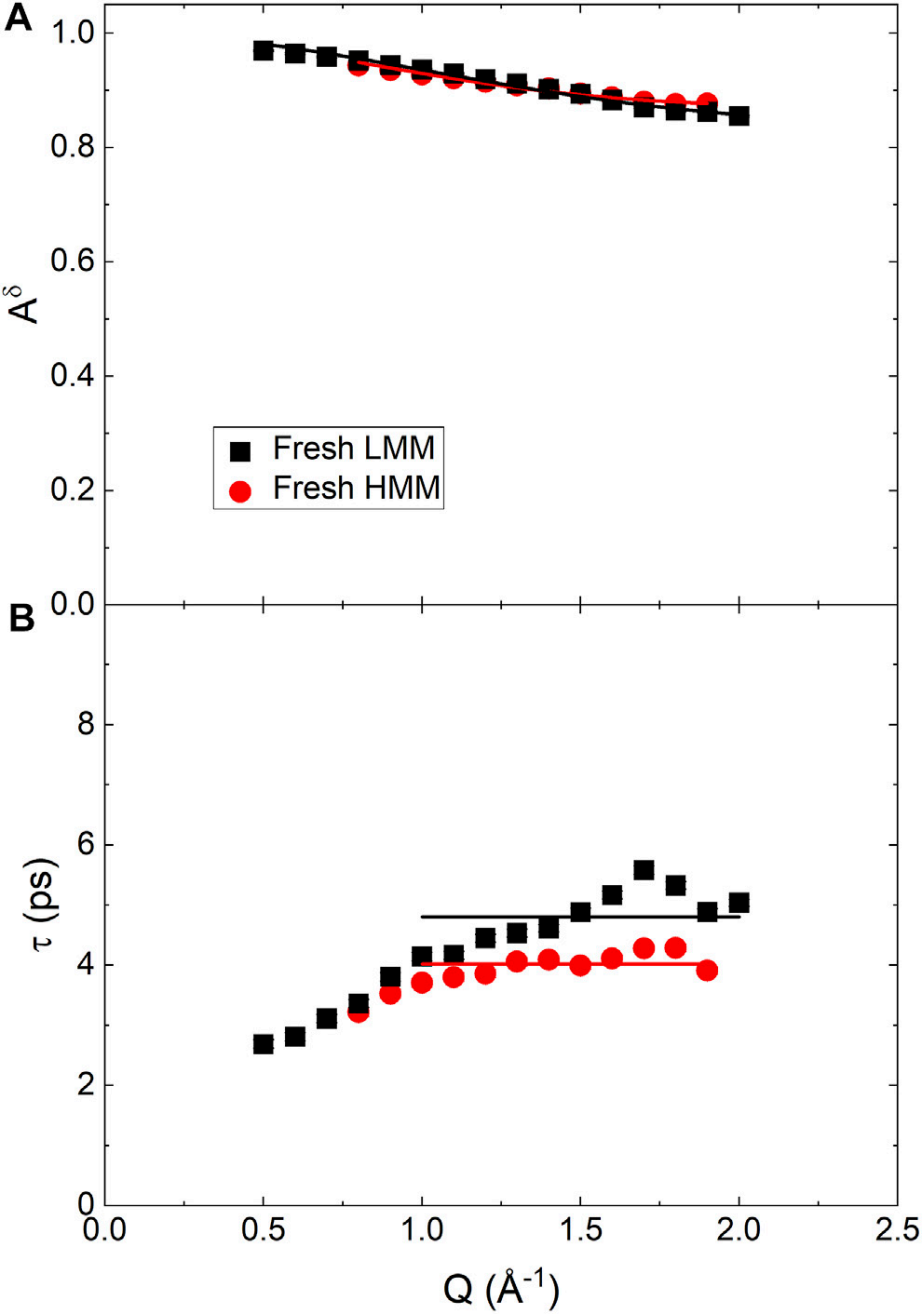
*Q* dependence of the best fitting parameters obtained from the QENS spectra of fresh low and high molecular mass pyrolytic lignin fraction using Eq. 20, with *β* = 1. **(A)** Continuous lines are the fitting according to Eq. 21. **(B)** Straight lines indicate the expected plateau value of *τ* for *Q* ≥1 Å^−1^.

Figure 8 reports the *Q* dependence of the fitting parameters. The *A* ^*δ*^ data were analyzed as the ones for BCO using Eq. 21; a mean relaxation time, $\bar{\tau }$ , was extracted as well. The obtained results are reported in Table 3. The main difference with respect to the BCO is the much larger immobile fraction, *A* ^imm^. The time scale of the motions are faster than the ones found in BCO which indicates that the dynamics observed originates mostly from local rearrangements of the chain conformation. The results are consistent with what reported in hydrated polymer systems (Noferini et al., 2019). Please, note that the fact that no significant stretching of the ISF was observed is attributed to the small fraction of mobile hydrogen atoms and the consequent difficulty in obtaining an accurate modeling.

## 4 Conclusion

BCOs hold tremendous potential as a novel renewable energy source. However, their reactivity partially limits the possibilities of their practical use. Gaining molecular insights on the properties of these systems might be the key to improve their applicability. However, from a physical chemical point of view, BCOs are extremely complex materials for their heterogeneity in molecular composition and large interface area of the diverse constituents. In this regard, they represent a practical example of widespread presence of interfacial water in nature. This paper reports the results of an investigation of the microscopic structure and dynamics of BCOs and their lignin components. The solid fraction, composed of mesoscopic carbonaceous aggregates, grows significantly with aging as evidenced by SANS. However, the changes in the microscopic dynamics of the interfacial liquid phases are more subtle. The dynamics of the hydrogen atoms, both in the solid and liquid phase, is limited to local re-orientation and conformation changes of the order of 1 to 2 Å. The timescales of the motion, around room temperature, are comparable to the ones of deeply supercooled bulk water or interfacial water in curing cement. This mobility is however required for the aging of BCO and elastic neutron scattering measurements suggest that storing the samples at ≈ 220 K might suppress its intrinsic reactivity.

## Note

Throughout the paper, error bars and uncertanties of the raw data represent one standard deviation, and error bars of the fitted parameters represent one standard deviation.

## Data Availability

The datasets presented in this study can be found in online repositories. The names of the repository/repositories and accession number(s) can be found below: https://www.ncbi.nlm.nih.gov/, JAGFBX000000000 https://www.ncbi.nlm.nih.gov/, JAGFBW000000000. Raw data were generated at ILL and NCNR. The derived data that support the findings of this study are available from the corresponding authors.

## Footnotes

1 In QENS, the term immobile is commonly used to refer to atoms whose dynamics is much slower than the instrumental time window of the instrument, in this case extending to ≈20 ps.
